# Understanding How Green Space Naturalness Impacts Public Well-Being: Prospects for Designing Healthier Cities

**DOI:** 10.3390/ijerph21050585

**Published:** 2024-05-02

**Authors:** Adriano Bressane, Mirela Beatriz Silva, Ana Paula Garcia Goulart, Líliam César de Castro Medeiros

**Affiliations:** 1 Institute of Science and Technology, São Paulo State University (UNESP), São José dos Campos 12245-000, Brazil; ana.goulart@unesp.br (A.P.G.G.); liliam.medeiros@unesp.br (L.C.d.C.M.); 2 Civil and Environmental Engineering Graduate Program, São Paulo State University (UNESP), Faculty of Engineering, Bauru 17033-360, Brazil; mirela.silva@unesp.br

**Keywords:** urban green spaces, naturalness, mental health, urban planning

## Abstract

*Statement of problem*: Urbanization has brought significant advancements in human well-being; however, it poses challenges to urban green spaces (UGSs), affecting environmental quality and public health. *Research gap*: Previous studies have established the importance of UGSs for urban well-being but have not sufficiently explored how the naturalness of these spaces—ranging from untouched natural areas to human-designed landscapes—affects mental health outcomes in the context of developing countries, particularly Brazil. *Purpose*: This study aimed to bridge the research gap by investigating the relationship between the degree of naturalness in UGSs and mental health among residents of Brazilian metropolitan areas. *Method*: Data were collected through an online survey involving 2136 respondents from various Brazilian urban regions. The study used Welch’s ANOVA and Games–Howell post hoc tests to analyze the impact of UGS naturalness on mental health, considering depression, anxiety, and stress levels. *Results and conclusions*: The findings revealed that higher degrees of naturalness in UGSs significantly correlate with lower levels of mental distress. These results underscore the necessity of integrating natural elements into urban planning to enhance public health. *Practical implications*: Urban planners and policymakers are encouraged to prioritize the preservation and creation of naturalistic UGSs in urban environments to improve mental health outcomes. *Future directions*: Further research should explore the specific attributes of naturalness that most contribute to well-being and examine the scalability of these findings across different cultural and environmental contexts.

## 1. Introduction

As the world undergoes rapid urbanization, it brings with it not only advancements in human well-being, such as improved sanitation, security, education, energy access, healthcare, and transportation services [1,2], but also challenges that compromise the integrity of urban green spaces (UGSs). The encroachment on and reduction in UGSs have been linked to adverse outcomes for environmental quality, public well-being, and the overall quality of urban life, exacerbating biodiversity loss, air pollution, and heat island effects [3,4,5]. Despite the growing body of evidence, existing studies reveal the complexity of the relationship between UGS diminution and health impacts, necessitating a more detailed understanding of this interplay.

The current literature has further validated the connection between limited access to green spaces and increased stress levels, decreased physical activity, and poorer mental health, reinforcing the vital role of UGSs in urban life quality [5,6,7]. Gong et al. [7] explored the relationship between urban green spaces, nature connectedness, and biodiversity, shedding light on how high-biodiversity green spaces can enhance well-being and green space visitation, thus emphasizing the importance of understanding the multifaceted role of UGSs in promoting health.

UGSs serve essential ecological and social functions within urban ecosystems. Ecologically, they are instrumental in conserving biodiversity, facilitating gene flow, and offering habitats that enhance urban ecological health [8,9]. These spaces act as natural buffers, mitigating soil erosion and climate change effects [10]. Socially, UGSs provide crucial areas for leisure and recreation, acting as serene retreats from urban life where individuals can engage in physical activities and foster social connections [11,12]. The health benefits associated with regular interaction with UGSs are profound, including stress reduction, mental health improvement, and enhanced physical activity [13,14].

While UGSs are acknowledged for their ecological and social benefits, the recent literature, such as studies by Zielonko-Jung and Wróblewska [15], has begun addressing the need for more detailed research into the spatial and qualitative attributes of UGSs, such as their naturalness. The exploration of “naturalness” within UGSs is pivotal, recognizing them as a spectrum from untouched natural areas to human-designed spaces.

The concept of naturalness within UGSs varies widely across studies, reflecting the complexity of measuring and comparing them across urban contexts [16]. In this research, we define naturalness as the degree to which UGSs mirror natural ecosystems, unaltered by human intervention. This encompasses a spectrum from highly natural, minimally disturbed areas to human-designed spaces, such as gardens and public squares [17]. By examining the nuances of UGS naturalness, this study contributes to a deeper understanding of how urban naturalness can be optimized to enhance public health and well-being, aligning with sustainable and health-centric urban planning strategies.

This discussion is particularly relevant in developing countries, like Brazil, where diverse environmental, cultural, and socioeconomic factors uniquely influence the UGS–well-being nexus. Despite the growing body of research supporting the positive impacts of UGSs on public health, studies predominantly focus on high-income countries, limiting the applicability of findings across different global contexts [2].

This study aims to bridge this gap by exploring the relationship between UGS naturalness and well-being in Brazilian metropolitan areas, offering insights into the unique environmental, cultural, and lifestyle factors influencing this dynamic. To do so, the study is designed to address the following questions: (*i*) How does the degree of naturalness in UGSs impact the mental health of urban residents? (*ii*) How can the findings inform the design and management of UGSs to optimize health benefits in urban areas, particularly in developing countries?

By focusing on the nuanced impacts of UGS naturalness on health and well-being, especially in the underexplored context of emerging economies, this study seeks to provide actionable insights that are both globally relevant and locally applicable, contributing to the advancement of sustainable and health-centric urban planning.

## 2. Materials and Methods

Data were collected through a comprehensive online public survey during September 2023, engaging a total sample size of 2136 respondents. All participants reside within the São Paulo metropolitan region, which consists of a conurbation of 39 municipalities. This specificity in respondent demographics ensured no influence from coastal or forest regional variations, and there was no seasonal variation that could have impacted the mental distress conditions of the respondents. This survey was designed to meet the ethical requirements for human studies in Brazil, as evidenced by the approval process (process #58149622.3.0000.0077) (Table 1).

**Figure 1:**
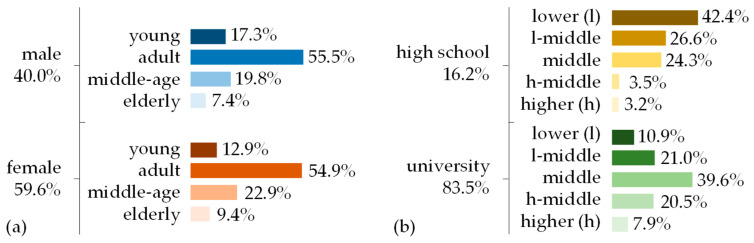
Sociodemographic profile of respondents: (**a**) age grouped by gender and (**b**) income grouped by education.

To ensure the recruitment strategy was both wide-reaching and targeted, the survey was disseminated via a multifaceted approach. The online questionnaire was extensively promoted and distributed through a range of online social media platforms, leveraging the vast networks and diverse user bases of these sites to attract a wide array of participants. Additionally, email invitations were systematically sent to a broad spectrum of educational institutions around the country. This strategy was used to tap into the rich demographic diversity within academic communities, thereby enhancing the representativeness of the sample (Figure 1).

To further ensure a diversified and representative sample of the metropolitan population and to bolster the validity of the findings, several additional measures were implemented. First, targeted outreach efforts were made in communities typically underrepresented in online surveys, including older populations and those in lower socioeconomic brackets, through partnerships with local community organizations and public libraries. Second, to mitigate potential biases associated with online survey participation, reminders were sent to increase response rates among those who might be less inclined to participate in online research initiatives.

The survey was organized into key sections. Initially, the opening section required participants to respond to questions derived from the Depression, Anxiety, and Stress Scale (DASS-21). This scale, extensively recognized for its application in both clinical and non-clinical populations worldwide [18], comprises 21 items, each scored on a Likert scale ranging from 0 (indicating the symptom was not experienced at all in the preceding week) to 3 (signifying the symptom was present). To translate these scores into measures of depression, anxiety, and stress, each domain within the DASS-21 is represented by 7 specific items. The total score for each domain is obtained by summing the scores of its corresponding items. For instance, scores for depression are calculated from responses to items focused on dysphoria, hopelessness, and devaluation of life, among others, while anxiety scores are derived from items assessing autonomic arousal and situational anxiety. Similarly, stress scores are obtained from items that measure difficulty relaxing and nervous arousal. Examples of questions in this scale include “I found it hard to wind down”, a common manifestation of stress; “I was aware of dryness in my mouth” and “I experienced breathing difficulty (e.g., excessively rapid breathing and breathlessness in the absence of physical exertion)”, which gauge physiological reactions often associated with anxiety; and “I couldn’t seem to experience any positive feeling at all” and “I felt that life was meaningless”, which reflect the profound emotional and existential challenges characteristic of depression.

Following this, in the second section, participants were asked to identify the locations where they had interacted with nature during the preceding week. This request was in line with DASS-21’s objective to evaluate mental well-being factors within a similar time frame. Based on these responses, locations in close proximity to wilder settings were classified as possessing higher naturalness, while others were deemed to have lower naturalness. As a more objective reference for classifying the naturalness of these locations, distinctive characteristics were considered. Participants were asked to describe the locations where they had interacted with nature in the preceding week, providing information that allowed for classifying the naturalness degree of environments encountered by the respondents. Thus, environments considered to have higher naturalness were those displaying a predominance of native plants; a diversity of wildlife; and the presence of natural bodies of water, rock formations, and other unmodified elements. Such locations were also characterized by natural sounds, such as birdsong, the noise of wind in the trees, or the flow of a river, and exhibited minimal presence of human constructions, such as buildings, roads, and other infrastructure. In contrast, environments classified as having lower naturalness predominantly featured cultivated vegetation, such as gardens with ornamental plants and well-maintained lawns requiring human upkeep, and displayed lesser diversity of animal species, often with frequent presence of domestic or urban-adapted animals. These environments possessed a significant presence of paved paths, benches, light fixtures, and other built structures, for example, areas designated for human recreation, such as playgrounds and sports courts, and were dominated by sounds of traffic, human conversations, and industrial or commercial activities. Additionally, these were locations where signs of pollution were evident, including the presence of litter and noise and visual pollution, as well as compromised air and water quality due to urban waste.

Prior to examining the direct and overarching effects of naturalness on well-being, any potential correlations with sociodemographic factors were considered and ultimately dismissed, as evidenced by the data presented in Table 2. This preliminary analysis ensured that the focus remained squarely on the intrinsic relationship between environmental naturalness and individual well-being.

Subsequent analyses were conducted using one-factor Welch’s ANOVA, followed by Games–Howell post hoc tests. This approach was predicated on the assumption of a sufficiently large sample size, which, according to the central limit theorem, ensures that the sample means are approximately normally distributed [19]. The use of Welch’s ANOVA, appropriate for comparing means across groups with unequal variances and sample sizes, alongside the Games–Howell post hoc test, which does not assume equal variances, underscores the methodological rigor applied in addressing the research questions. Following these analyses, the effect size was quantified using Cohen’s d ($d_{Cohen}$):

(1) $$d_{Cohen}=\frac{{µ}_{before}-{µ}_{after}}{\sqrt{\frac{(n_{before}-1){\sigma 2}_{before}+(n_{after}-1){\sigma 2}_{after}}{n_{before}+n_{after}-2}}}$$

where µ and σ are the mean and the standard deviation of the *i*th group, respectively.

Cohen’s d metric serves as a standardized measure of the magnitude of the observed effect, facilitating an objective assessment of the practical significance of naturalness for well-being. This step is crucial for interpreting the real-world implications of the findings, as it provides a scale-independent measure of the difference between groups. According to Funder e Ozer [20], a value around 5% (rho ≤ *d* < 7.5%) indicates a very small effect, around 10% (7.5 ≤ *d* < 15%) indicates a small effect, 20% (15 ≤ *d* < 25%) indicates a medium effect, and around 30% or greater (*d* ≥ 25%) indicates a large effect size.

To further elucidate the relationships between naturalness and mental distress scores, we conducted additional analyses using ordinal logistic regression. This approach enabled us to quantify the extent of the effect that naturalness has on depression, anxiety, and stress scores. Moreover, odds ratio coefficients were determined. An odds ratio greater than 1 indicates an increased likelihood of the event (higher levels of stress, anxiety, or depression) as the predictor increases, whereas an odds ratio less than 1 indicates a decreased likelihood as the predictor increases. All analyses considered a test power (1 − β) of 0.8 for a significance level (α) of 0.05, and a minimum detectable effect size (rho) of 6%. This analytical approach ensures that the conclusions drawn about the impact of environmental naturalness on well-being are both statistically valid and robust.

## 3. Results

**Figure 2:**
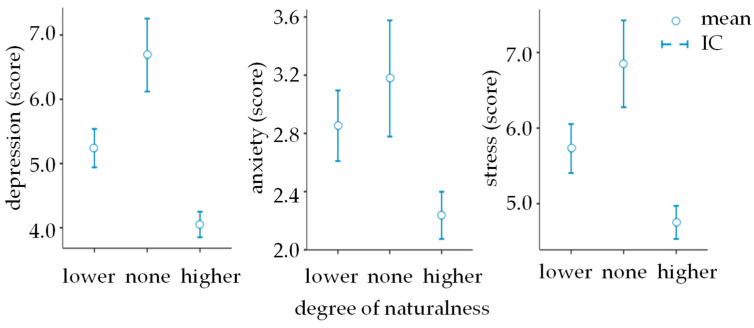
Association between degree of naturalness and mental health.

The findings from this research unequivocally support the hypothesis positing a substantial influence of environmental naturalness on well-being. Specifically, the data revealed a pronounced decrement in levels of depression, anxiety, and stress, with these reductions becoming more significant as the degree of engagement with wilder settings increases. This trend is systematically documented in Table 3 and visually illustrated in Figure 2, providing compelling evidence of the beneficial effects of natural environments on mental health.

Table 4 presents the regression coefficients, corresponding *p*-values, and calculated odds ratios based on the logistic regression analysis that explored the extent to which the naturalness of UGSs impacts mental distress outcomes.

For depression, the logistic regression yielded a regression coefficient of −0.584 (*p* < 0.001), indicating a statistically significant and inverse relationship with UGS naturalness. The corresponding odds ratio of approximately 0.558 suggests that for each unit increase in UGS naturalness, the likelihood of experiencing higher levels of depression decreases by approximately 44.2%. This finding underscores the therapeutic potential of more naturally designed urban green spaces in mitigating depressive symptoms.

In the case of anxiety, the regression coefficient was −0.340 (*p* < 0.001), again indicating a significant inverse association with UGS naturalness. The odds ratio of approximately 0.712 indicates that each incremental increase in naturalness is associated with a 28.8% reduction in the odds of higher anxiety levels. This supports the notion that natural elements within urban environments can serve as effective non-pharmacological interventions for reducing anxiety.

For stress, the regression analysis revealed a regression coefficient of −0.420 (*p* < 0.001), signifying a robust inverse relationship. The odds ratio of about 0.657 means that each unit increase in the naturalness of UGSs is correlated with a 34.3% lower probability of experiencing higher stress levels. This emphasizes the importance of integrating natural features into urban planning to alleviate stress among city dwellers.

The findings’ statistical significance underscores the pivotal role that natural environments play in fostering psychological well-being. These results highlight the imperative for policy interventions and the strategic design of UGSs that prioritize the integration of natural spaces within urban areas to address mental health challenges faced by city dwellers, as discussed later.

## 4. Discussion

Previous research, including studies by Topper et al. [21], Newby et al. [22], and Kotera et al. [23], consistently demonstrates the superior benefits of nature-related activities, such as forest bathing and natural-setting walks, in reducing depression and anxiety symptoms, over indoor activities, like gym workouts and sports clubs. This is in line with our findings that activities in natural areas significantly mitigate stress and anxiety symptoms more than those conducted in built-up areas, corroborating Song et al.’s [24] revelations that natural environments have a pronounced positive effect on mental health.

Although the positive effects of green spaces are well documented, the nuanced impact of varying degrees of naturalness has been less explored. Our study contributes to this discourse by illustrating that engagement with environments closer to natural ecosystems markedly improves mental well-being. This aspect has not been fully differentiated in earlier studies, such as those by Liu et al. [25] and Yigitcanlar et al. [26].

Recent research further illuminates this discussion. Gong et al. [7] have shown that biodiversity and nature connectedness are crucial for health and well-being, highlighting the importance of diverse and biologically rich urban green spaces. Xu et al. [27] demonstrate the mental health benefits of pocket parks, suggesting significant restorative effects on mental fatigue of even small green spaces. Additionally, An et al. [28] and Cardinali et al. [29] underscore the mental health benefits and importance of green space characteristics for social cohesion and mental health outcomes, respectively.

Our study found no significant effect on anxiety when comparing individuals with no contact with nature to those engaging with some nature (lower degree), described as environments predominantly characterized by cultivated vegetation, such as gardens with ornamental plants and well-maintained lawns, lesser diversity of animal species, significant presence of built structures, and a predominance of urban sounds and signs of pollution. This delineation is supported by previous studies, indicating that the perception and benefits of nature can vary significantly with the level of naturalness [30,31]. These findings underscore the importance of the qualitative aspects of urban green spaces in mental health interventions. A notable reduction in anxiety was observed only in environments with richer natural elements. This is partially supported by Yigitcanlar et al. [26], who noted positive mental health effects from urban park visits without differentiating the benefits by naturalness degree.

The effect size for stress demonstrates a significant correlation with the degree of naturalness, reinforcing the importance of quality in green space design. This finding is complemented by Elsadek et al. [32], who observed enhanced relaxation levels from natural elements in green facades compared to the relaxation levels in conventional built-up areas.

Mears et al. [33] highlighted the complex relationship between greenspace attributes and health indicators, including aspects such as signage, amenities availability, and cleanliness, yet without considering naturalness degrees. Similarly, the study by Allard-Poesi et al. [34] aligns with our findings, showing the varied impacts of different urban nature types on residents’ well-being.

The COVID-19 pandemic has underscored the importance of local contexts in the impact of green spaces on mental health, with Marques et al. [35] finding home gardens to be more beneficial than urban parks during self-isolation periods. This variability highlights the need for urban planning and public health policies to consider the complex nature of human–environment interactions. Liu et al. [36], Noe and Stolte [37], and Jarvis et al. [38] further support the significance of engaging with green spaces for health needs, with each study acknowledging the varied community needs and degrees of nature engagement. In summary, these studies collectively reinforce our findings regarding the effects of nature on well-being, emphasizing the critical need for considering these aspects in the design of greener and healthier cities.

## 5. Prospects for Designing Greener and Healthier Cities

The urbanization process in Brazil, characterized by an extensive gray infrastructure, contrasts sharply with our study’s findings, which underscore the critical need for integrating more natural settings into landscape and city planning to enhance well-being through nature engagement. Drawing from our research, which reveals significant improvements in mental health outcomes from interactions with environments closer to natural ecosystems, we introduce the following key prospects: (i) incorporating natural features in urban architecture, (ii) preserving and expanding urban forests, (iii) designing nature-infused urban environments, (iv) promoting equitable access to urban green spaces (UGSs), (v) launching public awareness campaigns, and (vi) collaborating on urban planning and health policies.

Inspired by the positive correlation between mental well-being and engagement with higher degrees of naturalness, architects and city planners should aim to seamlessly incorporate green roofs, vertical gardens, and other natural features into building designs. This approach is exemplified by the Eco-Courtyard project in São Paulo, which integrates green spaces within urban buildings, mirroring the success seen in Copenhagen, Denmark, for its sustainable urban planning that focuses on green element integration [39].

Our findings advocate for the preservation and expansion of biodiversity-rich areas and urban forests within Brazilian cities. This involves protecting existing natural habitats and promoting native vegetation through reforestation initiatives, similar to Curitiba’s approach to integrating green spaces and tree canopies into its urban fabric. This strategy aligns with actions taken in Portland, Oregon, emphasizing biodiversity and the well-being of residents through natural environments [40].

Reflecting our study’s emphasis on the mental health benefits of naturalness, urban planning across Brazil should prioritize the integration of green infrastructure, such as parks, greenways, and urban forests. These spaces should be universally accessible, encouraging models like Belo Horizonte’s development of urban parks and green corridors. Singapore, known as the “City in a Garden”, serves as a global benchmark for incorporating natural elements into urban planning [41].

Ensuring equitable access to nature-rich environments is paramount, as our study suggests that engagement with natural settings can significantly enhance mental well-being. Initiatives like Rio de Janeiro’s Green Doors project, which aims to make green spaces accessible to all residents, mirror efforts in Vancouver, Canada, to democratize access to nature and its mental health benefits [42].

To capitalize on our findings regarding the mental health benefits of nature, launching public awareness campaigns across Brazil is crucial. These campaigns should highlight the importance of conserving natural areas and encourage community engagement in nature-related activities. Rio de Janeiro’s Nature Guardians program and Melbourne, Australia’s citizen science programs exemplify how community involvement can foster a connection with nature [43].

Integrating nature-based interventions into mental health promotion strategies, as suggested by our study’s findings, calls for collaboration between urban planning and health policies in Brazil. Nature prescription programs, akin to San Francisco, California’s Park Rx, where time spent in nature is prescribed as part of treatment plans, could significantly benefit the Brazilian population [44].

By directly linking these urban planning prospects to the specific outcomes of our study, it is clear that prioritizing naturalness in urban environments, ensuring equitable access, and promoting the mental health benefits of nature interaction are crucial for enhancing the well-being of urban populations in Brazil and beyond.

## 6. Limitations and Future Research

The strengths of our study include a large and diverse sample size, which enhances the generalizability of our findings across various urban settings in Brazil. The use of a validated instrument, like DASS-21, also ensures the reliability of our measurements of depression, anxiety, and stress levels. Nevertheless, in recognizing the limitations of this study, it is essential to underscore that while our findings contribute significantly to the field of urban green space and mental health, they do not fully address the nuanced interactions that may exist between sociodemographic factors and diagnosis-specific mental health outcomes. Moreover, our study focused solely on the effects of naturalness, without considering its potential interactions with other aspects of urban design, social variables, or individual traits that might also influence mental health.

Further research endeavors could delve deeper into understanding how individual or community-level characteristics might impact these dynamics. It is important to acknowledge this perspective for future reference, ensuring a more comprehensive approach to urban planning and public health policy. Future studies are also crucial to discern the particular features of natural settings that have the greatest impact on well-being and to explore the underlying mechanisms by which natural environments affect mental health.

Looking ahead, it is crucial that future research focus on identifying the particular attributes of natural environments that most substantially contribute to human well-being. This exploration should aim to determine the applicability of these insights across various cultural and environmental landscapes, encompassing not only rural locales but also communities with distinct cultural identities. Exploring potential synergies between naturalness and other urban design elements, social factors, and individual traits could unveil more holistic approaches to enhancing well-being. Key factors such as local biodiversity levels, culturally specific practices regarding interaction with nature, prevailing economic conditions, and the existing infrastructural framework must be taken into account. These factors can present significant challenges in the direct transposition of findings from urban contexts to more diverse settings and require a deeper comprehension of the intricate relationship between natural environments and human well-being, especially within diverse urban settings.

Undertaking such comprehensive studies will deepen our understanding of the impact of natural features in green spaces on mental health across a wider spectrum of human environments. This detailed approach is essential for devising targeted strategies that effectively address the unique needs and attributes of different populations. This enriched perspective will not only bolster theoretical frameworks but also guide practical applications, fostering more resilient and adaptive environmental and community planning.

## 7. Conclusions

This study provides evidence that the degree of naturalness within UGSs significantly impacts the mental health and well-being of residents in Brazilian metropolitan areas. The data clearly illustrate that higher levels of engagement with natural settings—those closer to their natural ecological states—are associated with pronounced reductions in depression, anxiety, and stress among urban residents. These benefits are crucial in the context of rapidly urbanizing societies, where mental health issues are increasingly prevalent. The study’s methodology, using a comprehensive online survey and robust statistical analyses, ensures that these conclusions are both reliable and relevant across diverse urban contexts.

Moreover, the findings advocate for a nuanced approach to UGS design and management. It is not just the presence of green spaces that matters; their quality, accessibility, and the degree of naturalness also play pivotal roles in enhancing urban life quality. This study calls for a paradigm shift in how urban spaces are conceived, urging policymakers, urban planners, and health professionals to collaborate in creating greener, healthier, and more resilient urban ecosystems.

The global relevance of these insights cannot be overstated, especially as cities worldwide grapple with the challenges of urbanization and climate change. Brazil’s unique context, with its rich biodiversity and dynamic urban landscapes, provides a valuable model for integrating naturalness into urban development strategies.

In conclusion, our research highlights the critical role of UGSs in promoting mental health and well-being, emphasizing the need for strategic urban planning that incorporates naturalness as a core principle. The evidence presented underscores a clear mandate for creating more nature-infused urban environments, not only as a means to enrich biodiversity but also as a fundamental strategy for improving public health and enhancing the quality of urban life.

## Figures and Tables

**Table 1:** Sociodemographic profile of respondents.

Gender [Identity]	Age Group [Years]	Income [Min. Wage]	Education [Level]	Marital Status
Male 40%	Young [18, 25[ 15.7%	Lower) [0, 2[ 16.2%	Elementary 0.3%	Single 46.1%
Female59.6%	Adult [25, 45[ 55.2%	Lower-middle [2, 4] 21.9%	High-school 16.2%	Married 45.3%
Non-binary 0.4%	Middle-age [45, 60[ 21.0%	Middle ]4, 10] 37.0%	University 83.5%	Widower 0.9%
	Elderly [60, +∞[ 8.1%	Higher-middle ]10, 20] 17.7%		Divorced 7.7%
		Higher [20, +∞[ 7.1%		

**Table 2:** Potential interaction between naturalness and sociodemographic profile.

	*p*-Value *		
Interaction	Depression	Anxiety	Stress
Naturalness × gender	0.481	0.639	0.570
Naturalness × age	0.231	0.370	0.527
Naturalness × marital status	0.579	0.309	0.185
Naturalness × income	0.426	0.221	0.532
Naturalness × education	0.395	0.100	0.224

* Nonsignificant for *p*-value > 0.05.

**Table 3:** Effect of the degree of naturalness on depression, anxiety, and stress.

			None	Higher
Depression	Lower	Mean difference	−1.450 ***	1.190 ***
		$d_{Cohen}$	16.5%	15.7%
	None	Mean difference	-----	2.640 ***
		$d_{Cohen}$	-----	30.5%
Anxiety	Lower	Mean difference	−0.325	0.615 ***
		$d_{Cohen}$	5.07%	9.96%
	None	Mean difference	-----	0.940 ***
		$d_{Cohen}$	-----	15.1%
Stress	Lower	Mean difference	−1.120 **	0.977 ***
		$d_{Cohen}$	12.3%	11.9%
	None	Mean difference	-----	2.098 ***
		$d_{Cohen}$	-----	23.7%

* *p* < 0.05; ** *p* < 0.01; *** *p* < 0.001; gray = nonsignificant; yellow = small; green = medium; blue = large.

**Table 4:** Associations between naturalness and mental health outcomes.

Interaction	Coefficient	*p*-Value	Odds Ratio
Depression	−0.584	<0.001	0.558
Anxiety	−0.340	<0.001	0.712
Stress	−0.420	<0.001	0.657

## Data Availability

Ethical restrictions: Due to the nature of this research, participants of this study did not agree for their data to be shared publicly, so supporting data are not available.
